# Does the FDA have regulatory authority over adult autologous stem cell therapies? 21 CFR 1271 and the emperor's new clothes

**DOI:** 10.1186/1479-5876-10-60

**Published:** 2012-03-26

**Authors:** Michael Freeman, Mitchell Fuerst

## Abstract

FDA has recently asserted that many autologous cell therapies once considered the practice of medicine are in fact drugs. These changes began with the creation of new sections of 21 CFR 1271 and a subsequent one word change where the FDA, without public commentary, altered a single word in its regulatory language regarding cell and tissue based therapies that asserted the authority to classify autologous tissue as drugs. The bright line between medical care and drug production can be delineated in many ways, but a simple metric that defines the dichotomy is the consent status of the patient. In healthcare, a patient can either be consented individually for a medical procedure or exposed to an unconsented risk where regulatory assurances are already in place. These new FDA policies apply rules meant to keep drugs safe in a drug factory (unconsented mass production risks) to individually consented surgical procedures. We argue that there is little societal benefit to these changes and that they are already stifling medical innovation.

## Background

A recent editorial entitled, The King is Dead, Long Live the King: Entering A New Era of Stem Cell Research and Clinical Development by Ichim piqued our interest [1]. The piece described how the recent failure by Geron to commercialize embryonic stem cells for treatment of spinal cord injury might have signaled the decline of the commercial pursuit of such cells, and a change of market focus from embryonic to adult stem cells. We would argue that the primary reason that the cell therapy industry now finds itself in the midst of a financial funding crisis is in fact, due to unrestrained over-regulation by the FDA, rather than any lack of viability of the technologies. This over-regulation threatens to smother innovation in a nascent clinical movement that is already successfully translating promising therapies involving adult stem cells from the lab to the clinic. Although the FDA has never had the authority to govern and restrict the practice of medicine, this is precisely what they are now doing with regard to clinical uses of autologous stem cells. Until recently, this regulatory expansion has gone largely unnoticed, attracting little attention, with a few notable exceptions. To carry Ichim's regal metaphor a bit further, not only is the king dead, but the emperor has no clothes, and nobody's telling him he's naked.

## Discussion

Since it was first enacted in 1938, the U.S. Food, Drug, and Cosmetic Act (FDCA) has regulated medical drugs and devices based on basic public health concepts that recognize the differences between the practice of medicine and the mass production of drugs [2]. The regulation of mass manufactured and widely distributed medical products led to a revolution in healthcare that greatly increased public safety and unquestionably saved lives due to the reduction of unsafe products. The FDA regulation of transplant tissues through the Public Health Service Act has also advanced the public health through reduced disease transmission. This all changed in 2006, when the FDA, without public commentary, altered a single word in its regulatory language regarding cell and tissue based therapies that moved their focus from protecting the public from communicable disease transmission to asserting authority over virtually all therapies using *autologous *cells and tissue [3]. In effect, the Agency now claims regulatory authority over a broad category of medical procedures. Fifteen years ago a similar proposal brought forth an industry-wide tsunami of objections and complaints; today, there is nary a whimper [4].

The bright line between medical care and drug production can be delineated in many ways, but a simple metric that defines the dichotomy is the consent status of the patient. In healthcare, a patient can either be consented individually for a medical procedure or exposed to an unconsented risk where regulatory assurances are already in place. For example, an Individual Consented Risk (ICR) is defined as a medical procedure or therapy for which a patient is formally consented in order to ensure a thorough understanding of the risks and possible benefits of the care. An example of ICR is a cardiac surgery for which the health risk may be extreme and the benefits difficult to quantify. In this situation the onus is on the patient to make an informed decision, after full disclosure of the best estimate of risk of the procedure by the physician, whether to undergo the procedure. In contrast with ICR, Mass Production Risk (MPR) is not associated with formal patient consent, as there is a general assumption on the part of the prescribing physician as well as the patient that the risk associated with the use of medicine that has passed through regulatory oversight is acceptably low. In the U.S. the FDA is the entity that provides public assurance that certain minimal standards (i.e. purity, potency, efficacy, and safety) have been met before a drug can be legally introduced to the market.

Cellular medicine is a rapidly growing trend in healthcare that finds itself at the confusing crossroads of the regulatory concepts associated with ICR and MPR. An example of a widespread practice of cellular medicine is the use of autologous platelet rich plasma (PRP) to promote healing [5-7]. A less practiced but promising evolution of PRP involves the use of adult autologous stem cells (A-ASCs) for promotion of healing of injured tissue [8]. From a common sense perspective, it should be obvious that both of these autologous therapies are associated with ICR rather MPR, in that there is no mass production or distribution involved, the risk of procedure to the individual patient can only be estimated by the clinician who is providing the therapy, and it is up to the patient to weigh and ultimately accept the risk and provide consent for undergoing the procedure. This sort of common sense is absent in the FDA's adoption of 21 CFR 1271 in 2006, the rule that made a patient's own cells subject to the same regulation as mass produced drugs.

This movement away from common sense began in the 1990's when the FDA first signaled an interest in regulating the use of autologous cells [4,9-11]. The administrative records of the hearings from this time show that many large and highly credible organizations spoke out in opposition to this proposed intrusion into the practice of medicine. The list of organizations that objected to the FDA's proposed rule change included the American Red Cross, the American Society for Clinical Oncology, and the Society for Assisted Reproductive Techniques, among others. Many of the groups noted that the FDA was only granted authority by the U.S. Congress to regulate *allogeneic *tissue transplants in order to control communicable disease transmission, and that the Agency had no authority to regulate human cells of any type like mass produced prescription drugs. The most vocal opponent to these proposed regulations was the ASCO, who stated:

• *"ASCO objects in the strongest terms to FDA's proposed regulation of stem cell transplants. This misguided proposal is unnecessary... and exceeds FDA's legal authority*.

• *"...stem cell transplants are medical procedures. Their use is the practice of medicine, not the manufacturing of a drug as FDA asserts*.

• *"A striking aspect of FDA's proposal to regulate stem cell procedures is the virtual absence of any justification for the initiative"*.

• *"The FDA should not regulate stem cell procedures undertaken within an institution... or in any other setting where the cells are procured from a donor for a preselected recipient under the direction of physicians caring for these patients.... The proposed approach is a threat to good medicine and should not be adopted."*

Despite these industry concerns, by 2005 the Agency had completed publically announced changes to its regulations that classified "more than minimally manipulated" *allogeneic *cells the same as prescription drugs. This regulatory change at least made some sense, since allogeneic cells could be grown in large bioreactors and distributed to the public like a mass produced drug product. The FDA did not stop at allogeneic cell therapy in their bid to control all cellular therapy; in 2006, the FDA "clarified" 1271, asserting that the rule applied to *all *human tissue and cells, which meant that it now applied to autologous cells and tissue as well. This was accomplished with the alteration of a single word: "into ***another ***human" was changed to "into ***a ***human."^a ^For the first time, by nothing more than semantic sleight of hand, the agency bequeathed itself authority over a broad group of medical procedures and their attendant ICR risks. Recent decisions by agency show that it isn't yet done with expanding its regulatory authority into the practice of medicine.

Since 2006 the FDA has made several public statements regarding their intent to regulate the autologous use of human cells and tissue, with an ever-widening number of therapies being caught in their regulatory dragnet. As an example, in 2008, the FDA sent a notice to Regenerative Sciences, a medical practice that utilized expanded bone marrow derived mesenchymal stem cells for autologous orthopedic use [12]. Despite the medical procedures involving only ICR, the FDA asserted that their drug mass production rules applied to the practices at the clinic. In fact, the Agency's actions directed at Regenerative Sciences appear to only be the FDA's foot in the door of medical practice regulation, as long as the practice involves use of autologous tissue or cells.

As an example, the Agency has recently made a worrisome assertion about the processing of adipose tissue, a medical practice that has been around for more than 100 years [13]. The Agency's Tissue Reference Group (TRG) has issued a statement in which they claim that any isolation of the stem cell rich fraction of fatty tissue for orthopedic use (the stromal vascular fraction or SVF) by a physician for his or her own use in patients equates to the manufacture of a drug, even if that tissue is processed at the bedside [14]. In addition, the same TRG has also recently issued the same edict for the use of bedside processed SVF for breast reconstruction, claiming this procedure is also a drug [15]. While the same FDA arguments are used in this letter, more worrisome is that the agency claims that adipose stem cells taken from adipose tissue and used to reconstruct adipose breast tissue may be non-homologous. Since the agency's own definition of homologous is, "[cells that] perform the same basic function or functions", it's unknown what logic drove this decision [16]. These assertions will immediately restrict the medical practices of hundreds of U.S. physicians who use SVF to assist in healing, as no clinical practices can meet the stringent regulations that are applied to drug manufacturing. In addition, these decisions will only hamper medical innovation without measurably improving public safety.

To illustrate how poorly thought out the language in 1271 is, consider a recent publication in which it was reported that the magnetic field fluctuations of a routine MRI changes the biologic characteristics of stem cells [17]. These authors demonstrated that the magnetic fields induced by diagnostic MRI can alter fundamental biologic characteristics of stem cells such as gene expression and bone forming ability. Thus if, as is common surgical practice, an orthopedic surgeon creates a dilute autologous stem cell mix using a bone marrow aspirate, places the mixture into a patient's spine to promote fusion, and then orders an MRI post-procedure, the stem cells will become more than minimally manipulated. Whatever the FDA intended when they slipped new and greatly expansive language into 21 CFR 1271 in 2006, it is doubtful that they intended to shut down or restrict many commonly practiced and effective medical therapies that have nothing to do the mass manufacture and interstate of transport of drugs. It is even more doubtful that the U.S. Congress ever intended to the FDA to have such broad power over the practice of medicine.

Should there be concern that the FDA will continue to expand their attempts to regulate the practice of medicine? The FDA has recently demonstrated that the answer to this question is an emphatic YES. The aforementioned action against Regenerative Sciences spawned a series of suits and countersuits that are still in litigation. As part of this process, the Court hearing the cases recently issued an "Order to Show Cause" to the FDA. The order stated that, since Congress had only authorized the FDA to classify a substance that affected the body through "chemical action" as a drug, the Agency needed to clarify its position as to why a patient's own stem cells could be considered a drug [18]. The Agency's response to the order was dismaying; they responded with: "*When living cells interact with their environment to mediate repair of and/or regenerate damaged tissue, they do so by chemical action*." Since all living cells produce chemicals to interact with their environment, the Agency has made it clear that they believe that they have the authority to classify any living organism as a drug!

Is it reasonable for the FDA to assert that there is any benefit to imposing MPR regulations on medical procedures involving ICR? Medical therapeutic innovation comes to the public via two pathways; the slower big pharma drug pathway, and the substantially faster physician practice/discovery pathway. The pharma pathway has the advantage of producing high quality data from randomized controlled trials in support of a new therapy, but the disadvantages of inflexibility and glacially slow laboratory to clinical translation. In comparison, the physician practice pathway has the disadvantage of reliance on lower quality data (beginning, in all cases, with anecdotal experience), but the advantages of flexibility and a shorter time from discovery to clinical implementation. Physicians typically publish smaller studies that are reactive to problems encountered in daily clinical practice, and result from the rapid adoption of new therapies that appear to be effective in a semi- or uncontrolled setting. The pharma pathway has the potential to produce societal benefit in the form of increasingly effective and safer prescription drugs, but it is a far from foolproof system associated with a laundry list of unsafe drugs that were initially hailed as medical marvels (Vioxx, fen/phen, propoxyphene, Avandia, Baycol, *inter alia*) but later found to have unacceptable rates of serious, sometimes fatal complications. The physician pathway has also produced major medical advancements and benefits to public health such as modern in-vitro fertilization and almost all modern surgical procedures. To be sure, there have been major missteps along the way as well (bloodletting and most lobotomies, hysterectomies, and radical colectomies, to name just a few), but the medical profession and the bodies that provide education, licensure, regulation, and peer review tend to rapidly self-correct for harmful practices. The imposition of regulations meant to minimize MPR on the pharma pathway makes sense, as this pathway results in products meant for mass distribution. In contrast, the attempt to shoehorn medical practices into an MPR regulation model, as the FDA has done with autologous stem cell therapies, will produce no benefit to society, and harm public health by depriving society of the benefits of physician-initiated medical innovation.

Ultimately, the FDA's assertion that certain clinical or laboratory processes transform autologous cells into drugs is not based in a scientific risk analysis, and goes far beyond the Agency's mandate to protect the public from the risks associated with the mass production of drugs or devices. It certainly begs the question of how far the "medical procedure vs. drug" line may get redefined in the future. Are there compounded drugs that will be assigned MPR status? What about fertility culture procedures, such as a 5-day blastocyst transfer? If the therapeutic culture of a human being from two gametes isn't "more than minimal manipulation" of those cells then it's hard to imagine what is. Will surgical procedures have to someday comply with drug manufacturing guidelines?

All of these regulatory decisions have significant economic consequences for society. For example, medical care and drugs have different requirements for safety. An apt example that demonstrates the societal cost associated with imposing MPR regulations on ICR is applying drug factory Good Manufacturing Practices (GMP) to a hospital surgical procedure. GMP regulations are strict guidelines that ensure a minimum level of safety when millions of doses of a drug are manufactured, but are not used in a hospital setting. For example, if GMP regulations were applied to a hospital setting they would require that a hospital operating room (O.R) have a separate quality assurance program as does a major pharmaceutical factory. All surgical supplies would need to be separately validated to confirm the already FDA regulated manufacturer's claims. For example, sterile medical gases already regulated by the state would have to be again tested by the hospital. In addition, every surface in the operating room would have to be swabbed for microbial, viral, and fungal contamination on a regular basis and the FDA regulated cleaning solution claims separately validated in expensive testing performed by the hospital staff. In addition, each patient's wound would also have to be swabbed and sent for microbial, viral, and fungal contaminants before completion of the surgery. Operating room air handling would also have to be upgraded to GMP standards, costing hundreds of thousands of dollars per O.R. In addition, while surgeons are used to making adjustments in technique to fit the patient, each change in process would require a separate Standard Operating Procedure change control process. As a result, surgeons would be unable to alter the procedure to fit the patient. In addition, every step in the surgery would have time limits that would be strictly monitored and recorded and when any step exceeded timed parameters the surgeon would be written up by a quality assurance manager as having exceeded "processing parameters". While all of these changes may make good sense to protect the nation's drug supply from contamination, they make little sense when applied to an operating room and would also financially hobble an already expensive medical care system.

## Conclusion

The FDA has a long and important history of safeguarding the health of the U.S., but always in the context of its mandate to regulate medical products that carry with them risks associated with mass production. For the most part, the Agency usually gets it right; however in altering that single word of 21 CFR 1271, the FDA has overstepped its mandate, and through over expansive regulation is threatening to cripple medical innovation. Their most recent interpretation of 21 CFR 1271 indicates that the FDA is in need of a course correction. It's time that someone has the courage to tell the Emperor about the problems with his new wardrobe.

## Endnote

^a^Thus, section 1271.3(d) now states that "[h]uman cells, tissues, or cellular or tissue-based products (HCT/Ps) means articles containing or consisting of human cells or tissues that are intended for implantation, transplantation, infusion, or transfer into *a human *recipient." 21C.F.R. § 1271.3(d) (April 1, 2006) (emphasis added). *See also*, 21C.F.R. § 1271.3(d)(1) (April 1, 2010).
